# Comparative study of core needle biopsy and fine needle aspiration in the treatment of metastatic lymph nodes guided by contrast-enhanced ultrasound

**DOI:** 10.12669/pjms.38.6.5471

**Published:** 2022

**Authors:** Wei-na Mu, Jian-heng Li, Ying Liu, Hui-qing Liang, Xin Liu

**Affiliations:** 1 Wei-na Mu, Department of Ultrasound, Baoding No.1 Central Hospital, Baoding 071000, Hebei, China, Hebei University, Baoding 071000, Hebei, China; 2 Jian-heng Li, Hebei University, Baoding 071000, Hebei, China; 3 Ying Liu, Department of Ultrasound, Baoding Baoshihua Oriental Hospital, Baoding 071051, Hebei, China; 4 Hui-qing Liang, Department of Ultrasound, Baoding No.1 Central Hospital, Baoding 071000, Hebei, China; 5 Xin Liu, Department of Ultrasound, Baoding No.1 Central Hospital, Baoding 071000, Hebei, China

**Keywords:** Lymph node, Needle biopsy, Contrast-enhanced ultrasound

## Abstract

**Objectives:**

To compare the diagnostic efficacy of fine needle aspiration (FNA) and core needle biopsy (CNB) for metastatic lymph nodes guided by contrast-enhanced ultrasound (CEUS), and to provide reference for clinical selection of puncture methods.

**Methods:**

A total of 168 patients who were admitted to Baoding No.1 Central Hospital from June 2020 to January 2021 and required puncture of the diseased lymph nodes were included. Seventy six patients were guided by conventional ultrasound, of which 37 received FNA and 39 received CNB. 92 patients were guided by CEUS, of which 41 received FNA and 51 received CNB. The diagnostic accuracy of FNA and CNB guided by conventional ultrasound and CEUS was compared, and the sensitivity, specificity, positive predictive value, and negative predictive value of FNA and CNB in the diagnosis of metastatic lymph nodes guided by CEUS were further compared.

**Results:**

The diagnostic accuracy of FNA and CNB guided by CEUS were higher than that guided by conventional ultrasound, with a statistically significant difference (P<0.05). The sensitivity, specificity, positive predictive value, and negative predictive value of FNA and CNB in the diagnosis of metastatic lymph nodes were 95.0%, 95.2%, 95.0%, 95.2%, 100%, 100%, 100%, 100%, respectively, with statistically significant differences (P>0.05).

**Conclusion:**

CEUS can guide puncture and improve diagnosis accuracy. No statistical difference can be seen in the diagnostic efficacy of CNB and FNA for metastatic lymph nodes, CNB can provide more diagnostic information, while FNA can replace CNB for metastatic lymph nodes adjacent to blood vessels and difficult to operate.

## INTRODUCTION

Cervical lymphadenopathy is an unusual manifestation of a great many pathological processes, and it is essential to accurately diagnose and distinguish its benign or malignant. Cervical lymphadenopathy is commonly seen as self-limited diseases, lymphoma, tuberculosis and metastatic cancer.^1^,^2^ CEUS technique may provide extraordinary detailed lymph node perfusion by entering the blood circulation of lymph nodes via microbubbles, and diffuse and partial changes of lymph nodes can be shown even in lymph nodes with a diameter of less than 1cm.^3^

Fine needle aspiration (FNA) is a common method to evaluate cervical lymph node diseases, but its diagnostic accuracy varies depending on the nature of the lymph nodes.^4^,^5^ In recent years, coarse needle puncture (CNB) has been considered as a more effective method to obtain enough material from lymph nodes to accurately diagnose the nature of the lymph node.^6^ However, FNA is superior to CNB in that it is easy to operate, with less damage to surrounding tissues, and can be applied to smaller lymph nodes that are closely related to the blood vessels in the neck; In contrast, CNB is relatively complicated to operate and has a higher probability of complications, and is not suitable for smaller lymph nodes.^7^ Consequently, the comparative advantages of diverse methods should be weighed to determine the optimum puncture method for cervical lymph nodes.^8^

In this study, CEUS technique was applied to the examination of diseased lymph nodes to guide lymph node biopsy, increase the positive rate of puncture, and compare whether FNA and CNB have significant differences in the diagnosis of metastatic lymph nodes, so as to provide reference for clinical diagnosis of metastatic lymph nodes.

## METHODS

Patients who were admitted to Baoding No.1 Central Hospital from June 2020 to January 2021 and required puncture of the diseased lymph nodes were selected. Inclusion criteria: All patients requiring pathological diagnosis of enlarged lymph nodes, with enlarged lymph nodes ≥0.5cm in length and relatively full shape, and who are willing to undergo puncture. Exclusion criteria: Patients with enlarged lymph node cystic or partial cystic changes; Patients with underlying diseases who cannot tolerate puncture; Patients who have doubts about puncture. Among the 168 eligible patients, 82 were males and 86 were females. 76 patients were guided by conventional ultrasound, 31 were males, and 45 were females, of which 37 received FNA and 39 received CNB; 92 patients were guided by CEUS, 51 were males, and 41 were female, of which 41 received FNA and 51 received CNB. All patients were informed of the procedure and signed informed consent.

### Ethical Approval

The study was approved by the Institutional Ethics Committee of Baoding First Central Hospital on July 10, 2020 (No. [2020]073), and written informed consent was obtained from all participants

### Apparatus & Methods

Apparatus: A Philips EPIQ 7 ultrasonic diagnostic apparatus was used, with an eL18-4 probe, equipped with Elasto settings and Contrast settings. Sono Vue was utilized as a contrast agent when it comes to CEUS.

**Fig.1:**
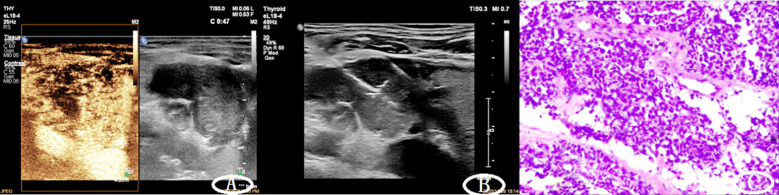
Male, 75 years old, with enlarged lymph nodes about 3.4×2.3cm in the IV area on the right side of the neck, and core needle puncture was performed. A: Comparison diagram of contrast-enhanced ultrasound and conventional twodimensional ultrasound; B: Ultrasound image of core needle puncture; C: Pathological results: Invasive carcinoma was found in the fibrous tissue, which was consistent with small cel l carcinoma combined with immunohistochemistry.

Routine two-dimensional examinations were performed on the lymph nodes of the diseased tissues to find out the morphological features such as the location, shape, size, boundary, internal echo, and posterior echo of the disease. Subsequently, a color Doppler ultrasound examination was performed, and the hardness of the lesion was judged by pressing the flexible button. Then the maximum section of the lesion or the section with the most abundant blood flow display was selected and switched to the contrast mode. Mechanical index (MI) was set at 0.06-0.14 in the contrast mode, and contrast agent was injected through the patient’s cubital vein with a pellet, while a timer was started. The position of the probe was fixed to keep the section unchanged, and the dynamic perfusion process of the lesion was observed continuously and in real time for no less than three minutes after injection. All images were stored in the built-in hard disk of the device. In case of unsatisfactory results of the first CEUS, a second injection of contrast agent can be performed within a safe dose to observe the CEUS performance of the lesion again. According to the location of lymphadenopathy of the patient, the optimum puncture site and puncture route were selected under ultrasound for core needle or fine needle puncture, and cells and tissue strips were taken respectively and sent for pathological examination. Ultimately, pathological results obtained by surgical resection or lymph node biopsy were regarded as the gold standard (Fig.1).

### Statistical Method

SPSS 20.0 software was adopted for statistical analysis. Measurement data were expressed as (mean ± standard deviation), independent sample t test was used, and counting data were expressed as number of cases. Chi-square test was utilized to compare the composition ratios of different lymph nodes in the FNA group and the CNB group. Fisher’s exact probability method was used to compare the sensitivity, specificity, positive predictive value, and negative predictive value of FNA and CNB for metastatic lymph nodes. P<0.05 indicates a statistically significant difference.

## RESULTS

The diagnostic accuracy of FNA and CNB guided by CEUS was higher than that by conventional ultrasound, with a statistically significant difference (P<0.05). Table-I and II.No statistical difference was observed in the incidence of metastatic lymph nodes between the FNA group and the CNB group guided by CEUS (P=0.692). Table-III.The sensitivity, specificity, positive predictive value, and negative predictive value of CNB were all higher than those of FNA, but P > 0.05 had no statistical significance. Table-IV.

**Table I:** Diagnostic accuracy of FNA guided by conventional ultrasound and CEUS.

Method		Final diagnosis		Diagnostic accuracy

		Metastasis	Other	
CEUS (n=41)	Metastasis	19	1	95.1
	Other	1	20	
Conventional (n=37)	Metastasis	18	4	75.7
	Other	5	10	
P value				0.021

**Table II:** Diagnostic accuracy of CNB guided by conventional ultrasound and CEUS.

Method		Final diagnosis		Diagnostic accuracy

		Metastasis	Other	
CEUS (n=51)	Metastasis	27	0	100.0
	Other	0	24	
Conventional (n=39)	Metastasis	22	3	76.9
	Other	6	8	
P value				<0.001

**Table III:** Comparison of the incidence of metastatic lymph nodes between the two groups.

Pathologic result	FNA(n=41)	CNB(n=51)
Metastatic lymph node	20(48.8%)	27(52.9%)
Other	21(51.2%)	24(47.1%)
X^2^ value	0.157	
P value	0.692	

**Table IV:** Comparison of FNA and CNB in metastatic lymph nodes.

Method		Final diagnosis		Sensitivity	Specificity	Positive predictive value	Negative predictive value

		Metastasis	Other				
FNA(n=41)	Metastasis	19	1	95.0%	95.2%	95.0%	95.2%
	Other	1	20				
CNB(n=51)	Metastasis	27	0	100.0%	100%	100%	100%
	Other	0	24				
P value				0.426	0.467	0.426	0.467

## DISCUSSION

CEUS is a new technique based on conventional ultrasound that forms a new acoustic interface in the blood circulation of target organs through micro bubbles, which can better display the tiny vessels and capillaries in lymph nodes and dynamically observe blood perfusion in real time, providing richer diagnostic information for the judgment of the nature of cervical lymph nodes.^9^,^10^ CEUS technique identifies changes in vascular structure in macrovascular, microvascular, and avascular areas by virtue of the difference in blood flow characteristics between normal and pathological tissues as signs of malignant infiltration.^11^ CEUS can improve the accuracy of differential diagnosis of benign and metastatic superficial lymph nodes by evaluating characteristic enhancement patterns.^12^ Such a technique showed greater sensitivity, specificity, and accuracy in distinguishing metastatic and reactive lymph nodes compared to pathological results.^13^ CEUS-guided needle biopsy is touted as increasing positive biopsy rates, reducing unnecessary repetitive work, and reducing complication rates.^14^

In this study, the diagnostic accuracy of FNA and CNB guided by CEUS was higher than that of needle biopsy guided by conventional ultrasound, which was similar to the results of Liang et al.^15^ Metastatic lymph nodes will have a characteristic enhancement pattern under CEUS, most of which are heterogeneous enhancement due to extensive necrosis and tumor cell infiltration. Specifically, the enhancement mode is a heterogeneous and centripetal enhancement mode, with low perfusion areas (tumor tissue) or non-perfusion areas (necrotic tissue) of varying sizes, followed by slow regression. The specificity, sensitivity and accuracy of traditional techniques in differentiating benign and malignant lymph nodes are lower than that of ceUS, suggesting that CEUS has a higher diagnostic effect. CEUS can not only guide puncture and improve the positive biopsy rate, but also predict the nature of lymph nodes. It can be used to observe the degree of lymph node enhancement, so that the lymph node perfusion area can be selected to avoid the non-perfusion area of lymph node for puncture biopsy, thus improving the success rate of puncture biopsy.^16^

The sensitivity, specificity, positive predictive value and negative predictive value of CNB in the diagnosis of metastatic lymph nodes were higher than those of FNA, but with no statistical significance. Previous studies have reported that CNB biopsy is more accurate than FNA in pathological results of lymph node biopsy.^17^ CNB can provide sufficient materials for routine histopathology, immunophenotyping as well as molecular examination, whereas FNA has obvious limitations in this respect. In other words, FNA cannot provide adequate materials for immunohistochemical analysis, which is challenging for the diagnosis of lymphoma and undifferentiated carcinoma. In cases where FNA fails to diagnose the nature of the lymph nodes, an open biopsy is required by a surgeon, although in this case the fascia may be damaged and the cancer may spread. However, the research results of Al Nemer A et al showed that FNA has a slight advantage over CNB in the accuracy of breast cancer diagnosis, and the differences are comparable, which is contrary to the results of this study.^18^ This may be due to the non-guided puncture operation used in the study of Al Nemer A et al., which also explains the importance of contrast-enhanced ultrasound.

Previous studies have showed that CNB had a higher sampling satisfaction rate and a higher incidence of complications than FNA by comparing fine needle puncture and core needle puncture in cervical lymph nodes. CNB puts forward higher technical requirements for physicians in terms of operation, which may be limited especially when the lesions are adjacent to blood vessels.^19^,^20^ In contrast, FNA has the advantage of being simple to operate, having few complications, and playing an important role in smaller lesions adjacent to blood vessels. Furthermore, CNB takes longer time to obtain results and is more expensive than FNA.^21^,^22^ CNB had higher sensitivity, specificity, negative predictive value and positive predictive value than FNA in metastatic lymph node biopsy, but with no statistically significant difference. For metastatic lymph nodes that are small in size and adjacent to blood vessels, FNA has the same diagnostic efficiency as CNB and can replace CNB with lower complications.

### Limitations of the study

It includes a small sample size .Further studies are needed to compare the diagnostic efficacy of CNB and FNA in other rare diseases to confirm our observations.

## CONCLUSION

CEUS can guide puncture and improve diagnosis accuracy. As indicated in the results of this study, no significant difference can be seen in the diagnosis of metastatic lymph nodes between FNA and CNB. CNB can provide more adequate diagnostic information and tissue immune information for pathological diagnosis, avoid open biopsy surgery and reduce the possibility of cancer spread. FNA can replace CNB by virtue of its easy operation, less pain to patients, and obvious advantages for small lymph nodes adjacent to great vessels. No obvious difference can be seen in the diagnostic efficacy of CNB and FNA for metastatic lymph nodes. CNB is recommended for most biopsy in clinical practice, but FNA is preferred for small lymph nodes adjacent to great vessels.

### Authors’ Contributions:

**WM** & **XL:** Designed this study, prepared this manuscript, are responsible and accountable for the accuracy and integrity of the work

**JHL & HQL:** Collected and analyzed clinical data

**YL:** Analysis of data, significantly revised this manuscript.
